# What determines violence among female sex workers in an intimate partner relationship? Findings from North Karnataka, south India

**DOI:** 10.1186/s12889-019-6673-9

**Published:** 2019-03-29

**Authors:** Prakash Javalkar, Lucy Platt, Ravi Prakash, Tara Beattie, Parinita Bhattacharjee, Raghavendra Thalinja, Kavitha D. L., Chaitanya AIDS Tadegattuva Mahila Sangha, Satyanarayana Ramanaik, Martine Collumbien, Calum Davey, Stephen Moses, Rachel Jewkes, Shajy Isac, Lori Heise

**Affiliations:** 1 Karnataka Health Promotion Trust (KHPT), IT Park, 5th Floor, #1-4, Rajajinagar Industrial Area, Behind KSSIDC Admin Office, Rajajinagar, Bangalore, Karnataka 560044 India; 2 Faculty of Public Health and Policy, London School of Hygiene and Tropical Medicine (LSHTM), 15-17 Tavistock Place, London, WC1H 9SN UK; 3 Center for Global Public Health, University of Manitoba, Winnipeg, Manitoba R3T 2N2 Canada; 4 Chaitanya AIDS Tadegattuva Mahila Sangha, Opposite Anupama Hospital, Mallamanagar, Mudhol, Bagalkot 587313 India; 5 South African Medical Research Council, 1 Soutpansberg Road, Pretoria, South Africa; 6 Johns Hopkins University, 3400 N. Charles Street, Baltimore, MD USA

**Keywords:** Female sex workers, Violence, intimate partner violence, Domestic violenc, Devadasi, India

## Abstract

**Background:**

Like other women in India, female sex workers (FSWs) frequently experience violence from their intimate partners (IPs)-a reality that increases their risk of acquiring HIV or other sexually transmitted infections. Less is known about the nature of these intimate relationships or what aspect of the relationship increases the risk of IP violence (IPV). We measured the prevalence and determinants of IPV on FSWs in the context of north Karnataka, India, characterized by high HIV-prevalence and extreme poverty.

**Methods:**

Overall 620 FSWs with an IP participated in a baseline survey conducted for an on-going cluster-randomised controlled trial aiming to evaluate the impact of a multi-level intervention on IPV reduction. We characterize the nature of intimate relationships and explored determinants of severe physical and/or sexual IP violence using univariable and multivariable analyses.

**Results:**

The median age of participants was 35 years with 10 years of duration in an intimate relationship. Though most relationships originated from a sex work encounter, 84% stated that IPs did not know they were currently practicing sex work. In past 6 months, the experience of emotional violence was 49% (95%CI:45.2–53.2), physical 33% (95%CI:29.5–37.1) and sexual violence 7% (95%CI:4.8–8.9), while 24% (95%CI:21.0–27.9) FSWs experienced recent severe physical and/or sexual violence from IPs. Factors associated with recent IPV included experience of physical and/or sexual violence from their clients in last 6 months (AOR 2.20; 95%CI: 1.29–3.75), sexual intercourse in the past 1 month when their IP was under the influence of alcohol (AOR 2.30; 95%CI: 1.47–3.59) and providing financial support to their IP (AOR 2.07; 95%CI: 1.28–3.34).

**Conclusions:**

The association between increased risk of violence and provision of financial support to an IP is indicative of gendered power dynamics as men remain dominant irrespective of their financial dependency on FSWs. Interventions are needed that address inequitable gender norms which makes FSWs tolerate violence even though she is not financially dependent on IP. Higher likelihood of violence in presence of alcohol use and FSWs’ previous experience of workplace violence linked to IPV call for strengthening the crisis management systems within community-based organisations that can address all forms of violence and associated risk factors.

**Trial registration:**

Clinical Trials NCT02807259

## Background

Female sex workers can face multiple, complex and inter-dependent health harms. The risk of HIV infection in low and middle-income countries is 12 times higher among female sex workers than women of the same age not engaged in sex work [1]. Violence against female sex workers is widespread originating from a range of perpetrators including intimate partners, police, pimps and paying partners [2, 3]. There is a growing body of evidence to show that exposure to violence among female sex workers is associated with many adverse health outcomes including: increased prevalence of HIV and sexually transmitted infections (STI); poor emotional health; increased alcohol or drug misuse; and reduced access to STI/HIV clinics [4–7].

The mechanisms through which violence adversely affects women’s health are complex and bi-directional. Violence may increase risk of HIV/STI transmission directly through forced unprotected sex. Evidence suggests that coerced sex is rarely protected and can result in injuries that increase the risk of transmission of STIs and HIV [8–10]. Exposure to violence can also lead to depression and low self-esteem, which in turn may lead to alcohol or drug use and reduced ability to negotiate condom use. This in turn can compound low self-esteem and emotional health problems [11]. Additionally, broader gender inequalities are key determinants of both STI/HIV transmission and violence among female sex workers, and violence often plays an important role in reproducing gender inequalities leading to higher risk of HIV/STI transmission [12, 13]. Gender inequalities that give men power over women increases the risk of violence against women, by reducing their ability to negotiate safe and consensual sex, and hindering women’s recourse to justice and help [14]. Evidence shows that men who are violent are more likely to have multiple concurrent partners, use condoms less frequently, have unprotected anal sex and report substance use [15]. All these factors have been linked to increased risk of HIV/STI transmission among female sex workers [16, 17].

Recent estimates suggest there are approximately 8,68,000 women in India who are currently engaged in sex work [18]. Corresponding estimates for Karnataka stands as 1,05,310 [19]. Sex work is closely linked to caste discrimination, poverty and gender inequality that pervades in much of India, with practices of underage marriage and dedication of young girls into sex work as part of religious traditions including the ‘devadasi’ system in northern parts of Karnataka [20]. Although, the devadasi system was made illegal in 1988, it is still one of the most common forms of traditional sex work in north Karnataka [21]. More than 90% of female sex workers in northern Karnataka come from Devadasi families and represent the most marginalised ‘scheduled’ castes or tribes [20].

Research documenting violence among female sex workers has in the majority focussed on client violence and not intimate partners [3]. There is however a growing body of evidence documenting the prevalence of intimate partner violence (IPV) among female sex workers that shows how in some settings it increases vulnerability to HIV and poor emotional health as much as violence from other perpetrators [22, 23]. Despite increasing awareness of the need to include intimate partners of female sex workers in research and interventions particularly in relation to HIV and STI programmes, and the links between violence and HIV/STI transmission, most violence prevention interventions for female sex workers focus on violence with clients, police or other actors relating to the organisation of sex work rather than IPV [24, 25]. It is essential to reduce violence experienced by female sex workers from all actors and to do this we need to understand the extent of exposure to IPV among female sex workers, the extent to which IPV affects health and how intimate relationships interact with the organization of sex work. This study is drawn from the baseline data of a cluster randomized controlled trial that seeks to evaluate the impact of a multi-level intervention to reduce violence from intimate partners among female sex workers in North Karnataka, in the context of high prevalence of HIV and extreme poverty [26]. In the absence of research focusing on the nature of intimate partner relationships among female sex workers, this paper examines the characteristics of relationships between female sex workers and their intimate partners and what aspects of the relationship determines IPV.

## Methods

In June 2014 we undertook a cross-sectional baseline assessment of female sex workers across 47 villages in Bagalkot district, north Karnataka. Eligibility criteria for participation included being older than 18 years and reporting an intimate partner in the last 6 months. Participants who had left their intimate partners in the 6 months preceding the survey were also included in the study. An ‘intimate partner’ (IP) was defined as husband, boyfriend, lover or a live-in partner who isn’t charged for sex. Most intimate partners of FSWs in this setting are married to other women and live with their families. All women were current sex workers at the time of recruitment.

A team of six female field workers recruited participants from a regularly updated list of eligible female sex workers maintained by the sex-worker collective community-based organisation (CBO) ‘Chaitanya AIDS Tadegattuva Mahila Sangha’, responsible for implementing the Samvedana Plus intervention [26]. All eligible female sex workers listed from 47 villages were approached for a face-to-face interview, and those who consented to participate were interviewed. Field workers received training on confidentiality, understanding of gender inequalities, violence and HIV to prepare them to respond appropriately to disclosures of violence and refer respondents to sources of support. Training of field workers covered all aspects of the study protocol, informed consent procedures and the survey tool.

The study was approved by the Institutional Ethics Committee of St. John’s Medical College and Hospital, Bangalore, India, and the Observational/Interventions Research Ethics Committee of the London School of Hygiene and Tropical Medicine. A community advisory board of female sex workers was set up to oversee all aspects of study design, implementation and consent procedures, and to address any adverse event that might occur from participating in the study. Since illiteracy is high among female sex workers in the region, verbal consent was accepted for participation, witnessed by counsellors supporting the intervention and study teams.

Field investigators completed pen and paper questionnaires during face-to-face interviews conducted in a private setting. Questionnaires involving closed-ended, pre-coded items were developed using, where possible, questions that had been used extensively in multi-centre studies conducted in resource-constrained countries [27]. Data were collected on: socio-demographic characteristics of both the female sex worker and her IP; sex work characteristics; characteristics of the intimate partnerships; the experience of violence; sexual risk behaviours; and exposure to HIV intervention programmes. In the case of more than one intimate partner (*n* = 7), information collected on the most important partner was included in all the analyses. All interviews were conducted in the local language, *Kannada.*

The primary outcome measure was the experience of “severe physical and/or sexual violence” from an intimate partner in the past 6 months. This was defined as experience of any act of moderate physical violence (pushed, shaken, thrown something, slapped or shoved) many times, and/or experience of any severe physical or sexual violence (hit, kicked, dragged, beaten, choked or burnt, threaten to use or actually used a knife, gun or any other weapon, physically forced to have sex against her will, forced to have sex under threat of violence or rejection, or forced her to do something degrading or humiliating to her) regardles of the frequency. Questions were adapted from the WHO (World Health Organisation) Multi-country Study on Domestic Violence and Women’s Health, and have been shown to have high internal consistency in different settings [27]. We focus particularly on the experience of severe physical and/or sexual violence since evidence from domestic violence research shows that this population is at increased risk of receiving sustained injuries and being in need of health care [28]. The prevalence of emotional violence in the last 6 month was also assessed using four items that inquired about humiliating, threatening, insulting actions and behaviours to scare or intimidate.

Potential covariates included in the analyses were individual and sex work characteristics, IP characteristics, and the characteristics of their relationship. FSWs individual and sex work level predictors included her age, literacy status (non-literate, literate), current marital status (never married, ever married), number of children (none, less than three, three or more), income other than sex work, average monthly income, place of solicitation (home, public places, phone/others), age at start of sex work, type of clients (occasional-clients who come once or a few times but are not known or recognized, regular- clients who are well known to female sex workers and regular visitors, occasional and regular), membership in a CBO, consistent condom use with clients in past 30 days, and experience of physical or sexual violence from clients in the last 6 months. IP level characteristics (derived from female sex worker responses) included information on the IP’s literacy status (non-literate, literate), current marital status (married, not married), occupation (cultivator, agricultural labourer, non-agricultural labourer, other work), caste (scheduled caste or tribe vs. other), whether the IP has children with other women, frequency of alcohol use (never, occasionally (less often), frequently (very often)).

Characteristics analysed at the relationship level were: was the IP a client before he became her IP, frequency of visit by IP (most frequent (daily/weekly), monthly (at least once in a month), less often (once in more than 1 month)), IP’s awareness of sex work profession, sexual intercourse with the IP in the last 7 days, IP under influence of alcohol during sex in past 1 month, provision of financial support to IP (for food, clothing or to fulfill his other needs), receipt of social support from the IP (defined as accompanying her when she goes out to the market, temple, shopping or any family functions), feeling afraid of the IP, refusing to have sex with the IP in the past 6 months, frequently receiving love and affection from the IP (receiving love and affection from the IP regularly), believing that her IP is unlikely to leave her and consistent condom use with the IP (defined as using condom in every sex act).

### Analysis

We examined univariable and multivariable associations between various co-variables and the outcome of interest (severe physical and/or sexual violence). Odds ratios (ORs) were used as the measure of association in all analyses and the likelihood ratio chi-square test was used to determine statistical significance. We followed a conceptual framework approach in conducting the multivariable analyses building on other conceptual frameworks that group variables according to their distal or proximal relationship to the outcome and drawing on ecological frameworks relating to IPV [29, 30]. We classified variables into different groups ranging from individual factors of female sex worker (socio-demographic and sex work characteristics) and intimate partners (characteristics of intimate partners) to relationship level factors (characteristics of the intimate partner relationship). This analysis was conducted in three stages. First, we assessed the association between each of the co-variables and the outcome. Second, individual variables from each grouping were included in separate subgroup models. Third, variables significant at *P* ≤ 0.10 in each of the multivariable subgroups were included in an overall multivariable model. This final model was adjusted for pre-hypothesised confounders: age and literacy status along with other factors found significant in the final model (*p* values ≤0.05). Stata V.14 (StataCorp LP, College Station, TX, USA) was used for all analyses.

## Results

### Sample characteristics

A total of 809 female sex workers were identified across the study sites. Of these, 620 (77%) participants completed the interview. The median age of participants was 35 years (interquartile range [IQR] = 28–40). The majority (89.8%) were Illiterate, were devadasi (96%) (Devadasi women are by tradition not allowed to marry a mortal man because they are considered “married” to one of the Hindu Gods), and 95% were consequently unmarried. Most (85%) reported having at least one child- with a median of two children (IQR = 1–3) per participant. Most (68%) female sex workers had children exclusively with their current intimate partners, 7% had children with current intimate partner as well as with other partners (clients or former intimate partners), and 11% had children exclusively with other partners (clients or former intimate partners). The majority (82%) had an alternate source of income other than sex work. The median duration of sex work was 19 years (IQR = 13–25). Three in four participants (76%) sold sex at their home, and the median number of clients per week was two (IQR = 1–3). More than half (54%) had both regular clients as well as occasional clients. More than half (61%) of women were members of the female sex worker community-based organization (CBO). These results are summarised in Table 1.

**Table 1:** FSW’s socio-demographic and sex work characteristics, and characteristics related to their IPs and intimate relationship

Characteristics	% or Median (IQR)	*n* = 620
Individual characteristics of sex workers		
Age		
Aged 25 years or below	11.9	74
Aged above 25 years	88.1	546
Median age of female sex workers (in years)	35.0 (28–40)	620
Illiterate^a^	89.8	557
Never married	95.2	590
Devadasi	96.1	596
Number of children		
No children	13.7	85
Less than three children	56.1	348
Three or more children	30.2	187
Median number of children FSWs have (Nos.)	2.0 (1–3)	620
Children with different partners		
Only current intimate partners	67.7	420
Current intimate partners and others	7.3	45
Only others	11.3	70
Sources of income other than sex work	82.3	510
Average monthly income		
Below Rs.3500	22.1	137
Between Rs.3500 to Rs.6999	60.5	375
Above Rs.6999	17.4	108
Median monthly income (in rupees)	4500 (3500–6000)	620
Sex work characteristics		
Place of solicitation		
Home	76.0	471
Public places	14.4	89
Phone/others	9.7	60
Age at first sex work		
Less than 15 years	38.7	240
15 years or older	61.3	380
Median age of first sex work (in years)	15 (14–16)	620
Duration of time in sex work		
Below 10 years	9.8	61
Between 10 to 19 years	41.0	254
Above 19 years	49.2	305
Median duration in sex work (in years)	19 (13–25)	620
Client volume per week		
below 3 clients	65.1	397
3 or more clients	34.9	213
Median no. of clients per week	2 (1–3)	620
Types of clients		
Only occasional clients ^b^	5.3	33
Only regular clients ^c^	41.1	255
Both occasional & regular clients	53.5	332
Member of community-based organisation (CBO)	61.2	379
Used condoms consistently^f^ with their clients (occasional and regular) in past 30 days preceding survey	91.8	560
Experienced physical violence from client in the last 6 months	15.2	94
Experienced sexual violence from client in the last 6 months	8.8	54
Experienced physical or sexual violence from clients in the last 6 months	16.3	101
Intimate partner (IP) characteristics^e^		
Median age of intimate partner (in years)	40.0 (35–45)	620
Illiterate^a^	66.9	415
Currently married	88.7	548
Occupation		
Cultivator	31.7	196
Agricultural labourer	32.8	203
Non-agricultural labourer	14.2	88
Other work	21.2	131
Belongs to Scheduled caste/Scheduled tribe	38.9	235
Has children with other women	71.6	444
Alcohol consumption		
Never	68.9	427
Occasionally	24.4	151
Frequently	6.8	42
Intimate relationship characteristics ^d^		
Belonging to different duration of intimate relationship		
below 5 years	14.0	87
between 5 to 9 years	23.7	147
10 or more years	62.3	386
Median duration of intimate relationship (in years)	10.0 (7–18)	620
Intimate partner was a client before he became intimate partner	63.8	394
Frequency of visit		
Most frequent (daily/weekly)	65.2	401
Monthly	27.0	166
Less often	7.8	48
Aware of FSW’s sex work profession	16.0	99
Had sexual intercourse with their intimate partner in the 7 days preceding the survey	58.4	362
Median number of times of sexual intercourse with the IP in the month preceding the survey	3 (2–6)	620
Intimate partner was under the influence of alcohol during sex in the month preceding the survey	24.0	149
FSWs provide financial support to their intimate partners	30.2	187
FSWs receive social support from their intimate partners	80.3	498
FSWs feel afraid of their intimate partners	50.5	313
FSWs refused to have sex with their intimate partners without using condoms in past 6 months	10.8	67
FSWs receive love and affection frequently from their intimate partners	75.3	467
FSWs believe that their intimate partner is unlikely to leave them	64.7	401
Used condoms consistently^f^ with their intimate partners	43.5	269

^a^ Who cannot read and write

^b^ clients who came only once or a few times, but FSW do not remember their faces or do not know them

^c^ clients FSW recognize well, who come to her repeatedly and she knows them

^d^ About seven FSWs reported having more than one intimate partner and another 12 FSWs reported that they had left their intimate partners in the last 6 months preceding the survey

^e^ As reported by female sex workers

^f^ Used condoms every time during sexual intercourse

### Characteristics of intimate partnerships

Almost all participants (97%) reported just one current intimate partner (IP) and only 1% (*n* = 7) more than one. Twelve (2%) women did not have a current intimate partner but had left an intimate partner within the last 6 months. The median age of intimate partners was 40 years (IQR = 35–45). Approximately 67% of intimate partners (IPs) were illiterate, and the majority (89%) were currently married. While almost all female sex workers were from scheduled caste or scheduled tribes, the majority of their IPs (61%) were from other castes. The majority of IPs (72%) had children with women other than their sex worker partner. Women reported that one quarter (24%) of IPs drink alcohol occasionally, and 7% drink frequently (Table 1).

The median duration of intimate relationships with the current IP was around 10 years (IQR = 7–18). Almost two-third (64%) of female sex workers met their intimate partner first as a client, however the majority (84%) said that their IPs are not aware of their current sex work profession. About one in three female sex workers (65%) met their IP regularly (daily or weekly) and 58% had had sexual intercourse with their IP in the past 7 days. Nearly one in four (24%) IPs were under the influence of alcohol during sex in the past 1 month. Almost all female sex workers (99%) said that they receive financial support from their IPs (not shown in the table) and about 30% of female sex workers reported providing financial support to their IPs while 80% received social support from them (defined as accompanying female sex workers to the market, temple, shopping or any family functions). Half of the women (51%) said they were afraid of their IP and two in three (65%) felt that their IP would not leave them (Table 1).

### Prevalence and frequency of different forms of violence

Table 2 summarises the findings on prevalence and frequencies of different forms of violence. The results on the severity of experiencing violence from the IPs shows that about 24% (95% CI: 21.0–27.9) of female sex workers experienced severe physical and/or sexual violence. One in three female sex workers (33%; 95% CI: 29.5–37.1) faced some form of physical violence from their intimate partners in the previous 6 months. The most common acts of moderate physical violence were being pushed, shaken or having something thrown at them. Hitting, kicking/dragging or beating were the most common acts of severe physical violence. In addition to this, 7% (95% CI: 4.8–8.9) experienced any form of sexual violence from their intimate partners in the prior 6 months. Nearly half of the women (49%; 95% CI: 45.2–53.2) reported experiencing emotional violence from the intimate partners during the same period. In most cases, the frequency of these violent acts was just once. However, among the acts of sexual violence, physically forcing sex was most frequently reported. Among acts of emotional violence saying or doing something to humiliate was most frequently reported.

**Table 2:** Prevalence and frequency of intimate partner violence in last 6 months among female sex workers

Forms of violence	Last six-month prevalence			
	Violence experienced	Frequency of events		
		Once	Few times	Many times
	n (%, 95 CI)	n (%)	n (%)	n (%)
Physical violence				
Any physical violence	205 (33.1%, 29.5–37.1)	108 (17.4)	81 (13.1)	16 (2.6)
Moderate physical violence:				
Pushed, shaken, or thrown something	146 (23.6%, 20.3–27.1)	74 (12.0)	58 (9.4)	14 (2.3)
Slapped or shoved	109 (17.6%, 14.6–29.8)	52 (8.4)	42 (6.8)	15 (2.4)
Any moderate physical violence	170 (27.4%, 23.9–31.1)	87 (14.0)	67 (10.8)	15 (2.4)
Severe physical violence:				
Hit with a fist that could hurt	102 (16.5%, 13.6–19.6)	65 (10.5)	24 (3.9)	13 (2.1)
Kicked/dragged or beating	97 (15.7%, 12.9–18.8)	43 (6.9)	41 (6.6)	13 (2.1)
Choked or burnt on purpose	9 (1.5%, 0.7–2.7)	5 (0.8)	1 (0.2)	3 (0.5)
Threatened to use or actually used a knife, gun or any other weapon	4 (0.6%, 0.2–1.6)	3 (0.5)	0 (0.0)	1 (0.2)
Any severe physical violence	146 (23.5%, 20.3–27.1)	84 (13.5)	47 (7.6)	14 (2.3)
Sexual violence				
Physically forced you to have sex when she did not want to	35 (5.6%, 4.0–7.8)	18 (2.9)	13 (2.1)	4 (0.6)
Used threats of violence or rejection to force her to have sex when she did not want to	21 (3.4%, 2.1–5.1)	11 (1.8)	6 (1.0)	4 (0.6)
Forced her to do something sexual that she found degrading or humiliating	7 (1.1%, 0.5–2.3)	7 (1.1)	0 (0.0)	0 (0.0)
Any sexual violence	41 (6.6%, 4.8–8.9)	22 (3.6)	13 (2.2)	4 (0.7)
Emotional violence				
Said or did something to humiliate her in front of others	260 (41.9%, 38.0–45.9)	182 (29.4)	61 (9.8)	17 (2.7)
Threatened to hurt or harm her or someone close to her	140 (22.6%, 19.3–26.1)	94 (15.2)	40 (6.5)	6 (1.0)
Insulted repeatedly to make her feel bad about herself	161 (26.0%, 22.6–29.6)	103 (16.6)	45 (7.3)	13 (2.1)
Did things to scare or intimidate her on purpose	145 (23.4%, 20.1–26.9)	79 (12.7)	49 (7.9)	17 (2.7)
Any emotional violence	305 (49.2%, 45.2–53.2)	160 (25.8)	124 (20.0)	20 (3.2)
Severity of violence				
Experienced severe physical and/or sexual violence	151 (24.4%, 21.0–27.9)	–	–	–

**Fig. 1:**
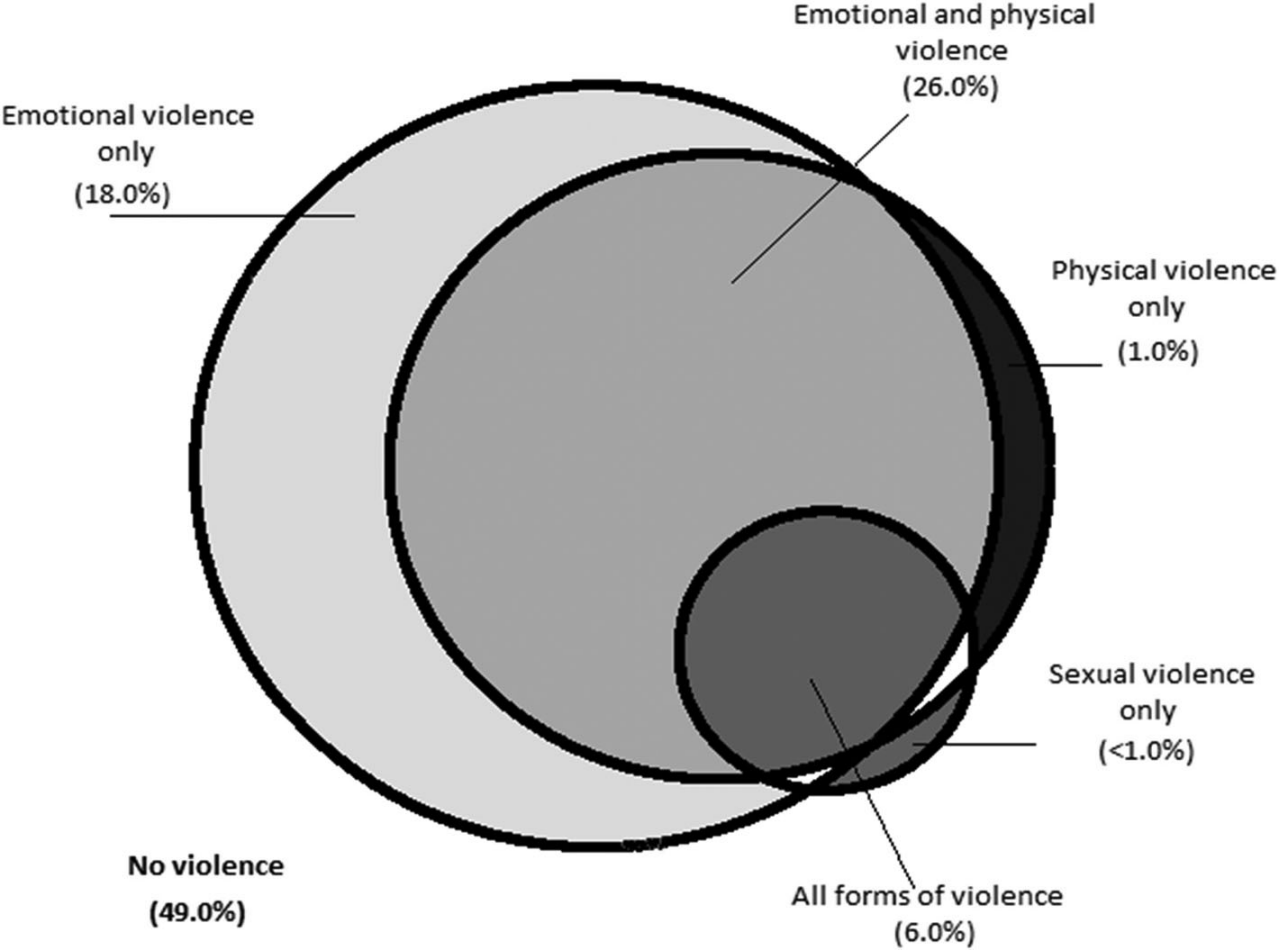
Proportional Venn diagram illustrating the overlapping between the different forms of violence

Figure 1 shows, the relationship between different forms of violence in the form of a proportional Venn diagram. For the most part multiple violence types were reported. Overall, about 26% of female sex workers reported the experience of both emotional and physical violence and 6% reported experiencing all three forms of violence from their intimate partners in the last 6 months. The most commonly occurring single form of intimate partner violence was emotional violence (18%).

Univariable associations indicated higher odds of severe physical and/or sexual violence among female sex workers aged 25 years or older compared to their younger counterparts (Odds ratio [OR] 2.49; 95% confidence interval [CI], 1.24–4.99), and among female sex workers who had children (with IP or others) (OR, 2.53; 95% CI: 1.08–5.92) (Table 3). Female sex workers had higher odds of severe physical and/or sexual violence if they reported an average monthly income of more than Rs.6999 (OR 2.28; 95% CI: 1.27–4.12), solicited clients in public places rather than receiving them at home (OR 1.87; 95% CI: 1.11–3.14), had been in sex work for 10 to 19 years (OR 3.21; 95% CI: 1.45–7.10), had three or more clients per week (OR 1.64; 95% CI: 1.11–2.42), and experienced physical or sexual violence from their clients in the last 6 months (OR 2.56; 95% CI: 1.59–4.11).

**Table 3:** Association between FSWs’ individual, sex work and relationship level predictors and their experience of IPV

Characteristics	Severe physical and/or sexual IPV in last 6 months		Unadjusted OR (95% CI)	Adjusted OR^a^ (95% CI)
	%	N		
Overall	24.4	620		
Individual characteristics				
FSW aged above 25 years (Ref- 25 years or below)	25.8	546	2.49 (1.24–4.99)^*^	2.31 (1.09–4.89)^*^
Literate (Ref- Non-literate)	33.3	63	1.64 (0.93–2.91)	1.98 (1.04–3.78)^*^
Ever married (Ref- Never married)	30.0	30	1.25 (0.55–2.80)	
Number of children (Ref- None)	18.8	85		
Less than three children	23.3	348	1.35 (0.74–2.47)	
Three or more children	28.9	187	1.77 (0.94–3.35)	
With whom FSW has children (Ref- No children)	18.8	85		
Only with IP	24.3	420	1.41 (0.78–2.56)	
IP and others	33.3	45	2.53 (1.08–5.92)^*^	
Others only	25.7	70	1.45 (0.67–3.14)	
FSW has any other source of income other than sex work (Ref-No)	24.7	510	1.08 (0.66–1.78)	
Average monthly income in rupees (Ref- Less than Rs.3500)	19.7	137		
Rs.3500 to Rs.6999	23.2	375	1.27 (0.78–2.08)	
More than Rs.6999	34.3	108	2.28 (1.27–4.12)^**^	
Sex work characteristics				
Place of solicitation (Ref- Home)	22.7	471		
Public places	31.5	89	1.87 (1.11–3.14)^*^	
Phone/others	26.7	60	1.29 (0.69–2.42)	
Age at start of sex work 15 years or above (Ref- Age less than 15 years)	25.3	380	1.18 (0.81–1.74)	
Duration in sex work (Ref- Less than 10 years)	13.1	61		
10 to 19 years	29.9	254	3.21 (1.45–7.10)^**^	
20 or more years	22.0	305	2.02 (0.91–4.48)	
Three or more weekly clients (Ref-Less than three weekly clients)	29.1	213	1.64 (1.11–2.42)^*^	
Type of clients FSWs have (Ref- Occasional clients)	24.2	33		
Regular clients	13.7	255	0.43 (0.17–1.04)	
Occasional and regular clients	32.5	332	1.44 (0.61–3.37)	
Member of community based organisation (Ref- Non-members)	21.6	379	0.69 (0.47–1.00)^*^	
Used condoms consistently with their clients in past 30 days (Ref- Not used)	23.8	560	0.63 (0.33–1.22)	
Experienced physical or sexual violence from clients in the last 6 months (Ref- Not experienced)	37.6	101	2.56 (1.59–4.11)^***^	2.20 (1.29–3.75)^**^
Intimate partner (IP) characteristics				
IP age 30 years or more (Ref- Age below 30 years)	25.2	563	1.98 (0.90–4.32)	
Having literate IP (Ref-Having non-literate IP)	24.9	205	1.08 (0.72–1.61)	
Having currently married IP (Ref-Having not currently married IP)	25.5	548	2.11 (1.05–4.26)^*^	
Occupation of IP (Ref- Cultivator)	26.5	196		
Agricultural labourer	24.1	203	0.85 (0.54–1.35)	
Non-agricultural labourer	19.3	88	0.65 (0.35–1.21)	
Other work	25.2	131	1.01 (0.60–1.69)	
IP from other caste (Ref-IP from scheduled caste or tribe)	26.0	369	1.28 (0.87–1.89)	
Has children with other women partner (Ref- No)	20.9	110		
Yes	25.9	444	1.32 (0.79–2.20)	
Don’t know	19.7	66	0.99 (0.46–2.15)	
Frequency of alcohol use (Ref- Never)	19.1	425		
Occasionally	26.5	151	1.46 (0.94–2.26)	
Frequently	66.7	42	9.26 (4.43–19.38)^***^	
Intimate relationship characteristics				
Duration of intimate relationship (Ref- Less than 5 years)	19.5	87		
5 to 9 years	22.4	147	1.17 (0.60–2.28)	
10 or more years	26.2	386	1.44 (0.80–2.59)	
IP was client before he became IP (Ref-No)	23.9	394	0.97 (0.66–1.42)	
Frequency of visit by IP (Ref- Most frequent (daily/weekly))	22.4	401		
Monthly	27.1	166	1.34 (0.88–2.05)	
Less often	27.1	48	1.25 (0.63–2.49)	
IP aware of FSW’s sex work profession (Ref- No)	15.2	99	0.47 (0.26–0.84)^*^	
Had sexual intercourse with IP in the last 7 days (Ref-No)	21.5	362	0.68 (0.46–0.98)^*^	
Number of sexual intercourse with IP in the last 1 month (Ref- Less than 3 times)	26.0	262		
3 to 4 times	20.9	139	0.69 (0.42–1.14)	
5 or more time	24.0	183	0.87 (0.56–1.35)	
IP was under influence of alcohol during sex in the month preceding the survey (Ref- No)	38.3	149	2.41 (1.60–3.62)^***^	2.30 (1.47–3.59)^***^
FSWs give financial support to IP (Ref- No)	30.5	187	1.43 (0.97–2.11)	2.07 (1.28–3.34)^**^
FSWs receive social support by IP (Ref- No)	21.9	498	0.53 (0.34–0.82)^**^	0.38 (0.23–0.65)^***^
FSWs feel afraid of their IP (Ref- No)	30.0	313	2.20 (1.50–3.22)^***^	1.94 (1.23–3.04)^**^
FSWs refused to have sex with IP in the past 6 months preceding the survey (Ref- No)	32.8	67	1.50 (0.86–2.61)	
FSWs frequently receive love and affection from their IPs (Ref- No)	22.7	467	0.65 (0.43–0.99)^*^	
FSWs believe that their IP is unlikely to leave them (Ref- No)	19.0	401	0.40 (0.27–0.58)^***^	0.46 (0.30–0.72)^**^
Used condoms consistently with their IPs (Ref- No)	24.2	269	0.95 (0.65–1.38)	

^*^p < 0.05; ^**^p < 0.01; ^***^p < 0.001. ^a^ Final model is adjusted for age, literacy, experience of physical or sexual violence from clients in the last 6 months, IP under the influence of alcohol during sex, sex workers providing financial support to IP, sex workers receiving social support from IP, sex workers afraid of their IP, sex workers believe their IP is unlikely to leave them

Similarly, having an IP who was currently married (OR 2.11; 95% CI: 1.05–4.26), who consumed alcohol frequently (OR 9.26; 95% CI: 4.43–19.38), or who was under the influence of alcohol during sex in past month (OR 2.41; 95% CI: 1.60–3.62) and feeling afraid of their IP (OR 2.20; 95% CI: 1.50–3.22) were associated with higher odds of severe physical and/or sexual violence among female sex workers. Decreased odds of severe physical and/or sexual violence was associated with IPs being aware of their partners’ sex work profession (OR 0.47; 95% CI: 0.26–0.84), having sexual intercourse with their IP in the last 7 days (OR 0.68; 95% CI: 0.46–0.98), the IP providing social support (OR 0.53; 95% CI: 0.34–0.82), love and affection (OR 0.65; 95% CI: 0.43–0.99), and female sex workers believing that their current IPs are unlikely to leave them (OR 0.40; 95% CI: 0.27–0.58).

After adjustment for confounders, increased odds of recent severe physical and/or sexual violence from an IP remained associated with experience of physical or sexual violence from a client in the last 6 months (AOR 2.20; 95% CI: 1.29–3.75). In addition, factors specifically related to the intimate relationship that remained siginificant in the multivariate model included sexual intercourse in past 1 month when IP was under the influence of alcohol (AOR 2.30; 95% CI: 1.47–3.59), providing financial support to the IP (AOR 2.07; 95% CI: 1.28–3.34), and feeling afraid of their IPs (AOR 1.94; 95% CI: 1.23–3.04). Receiving social support from an IP (AOR, 0.38; 95% CI, 0.23–0.65) and confidence that the IP would not leave them (AOR, 0.46; 95% CI, 0.30–0.72) remained significantly associated with reduced odds of recent severe physical and/or sexual violence from their intimate partners (Table 3).

## Discussion

We found high levels of severe physical and/or sexual violence (24%) within the last 6 months from intimate partners among female sex workers in Bagalkot district of Karnataka, India. Our study provides important new information showing that emotional violence from intimate partners is the most pervasive form of violence experienced in the last 6 months by FSWs (49%), followed by physical (33%) and sexual violence (7%). Findings clearly point to a substantial overlap between these different forms of violence, especially emotional and physical violence, suggesting that female sex workers in an intimate relationship are exposed to multiple forms of violence at a given point in time. The experience of physical or sexual violence from intimate partners was associated with increased risk of violence from clients during sex work, indicating a highly vulnerable population. Findings show that providing financial support to IP was associated with increased risk of violence reflective of broader gender inequalities that dictate that men who provide for women. Good communication, social support and trust were associated with reduced risk of IPV, showing that in context of long-term intimate partner relationships strategies to reduce intimate partner violence apply are the same across all populations irrespective of sex work.

Our findings build on other evidence that shows that female sex workers are highly vulnerable to sexual and physical violence from intimate partners as well as clients [5, 31]. Our findings provide important new evidence showing a high prevalence of emotional violence experienced by female sex workers and its intersection with physical violence. While levels of recent severe/moderate violence experienced by female sex workers are comparable to other reports of violence from intimate partners among female sex workers in the region [31, 32] measures of emotional, physical or sexual violence are far higher than in other low-income contexts – pointing to extreme vulnerability experienced by this population [6].

We found a clear association between intimate partner violence and increased risk of violence from clients. While this association has been evidenced elsewhere, including among street-based female sex workers in Vancouver [33] and Mexico [7], it provides important new information in the Indian context. This finding builds on previous research that has shown associations between experiencing physical or sexual violence from husbands and reduced condom use with clients as well as accepting more money for unprotected sex [31]. There is limited evidence on how intimate partners influence female sex workers’ interactions with clients, but emerging data suggest that female sex workers’ intimate relationships may influence their interactions with clients or vice-versa [7]. Qualitative evidence from drug-using female sex workers in Canada suggested that partners exercise control over female sex workers’ drug use, selection of clients, access to condoms as well as amount earnt per transaction [33]. More research is needed to understand the nature of this association in this context in order to design appropriate interventions.

The association between female sex workers’ providing financial support to their IP and increased risk of IPV has been shown in other low-income contexts [34]. Evidence from our qualitative work suggest that the desire for social acceptance lead women to frame their relationship as a marriage, with Devadasi increasingly relying on male partners to support them. When this financial commitment becomes too much for the intimate partner who often have other families to support (88% of male partners were married and 71% had children with other women) the Devadasi may take on this financial role in order to maintain the relationship (personal communication S. Ramanaik, 2018) [35]. Social expectations require men to be providers and women to take more modest care taking roles, conflict may arise when this role is reversed, compounded by the stigma arising from the money originating from sex work [32]. Traditional social expectations are more difficult to apply to sex workers and particularly Devadasi who traditionally cannot marry and are expected to look after their whole family [20]. It is also likely to reflect broader gender inequalities and gendered power dynamics, where men remain dominant even when financially dependent [34]. This is particularly acute within a society where domestic violence against women is generally accepted as the norm and with little recourse to legal support [36].

These baseline findings form part of a clustered-randomised controlled trial that will evaluate the ‘Samvedana Plus’ intervention designed to reduce violence and increase condom use within the intimate relationships of female sex workers in Bagalkot district of Karnataka. This comprehensive, multi-level intervention includes counselling for female sex workers and their IPs as couples and individuals and give strategies to reduce conflict and violence, as well as community level engagement in villages to question existing gender stereotypes and norms, and strengthening crisis management teams within the CBO to which female sex workers who experience violence can turn to for support and advice [26]. Causal pathways through which the intervention is hypothesized to work is that violence within intimate partner relationships can be reduced in the context of stronger, more openly communicative and gender equitable relationships [26, 37, 38]. Initial findings of this baseline data support this theory. We found social support (defined as being accompanied to the market or social and family functions) and security (not thinking a partner will leave) to be associated with reduced IPV. It is important to note that these indicators of social support can characterize a successful relationship in other non-sex working populations and indicate that in the context of long-term intimate partner relationships strategies to reduce intimate partner violence are the same irrespective of sex work. Other research has shown the benefit of intimate partnerships and how being treated as a person and not just a sex worker can foster feelings of inclusion [39]. At the same time when sex work related stigma and the discourse of blame, contempt and disrespect enters into an intimate relationship, often occurring alongside alcohol use, this can be more emotionally damaging that when it occurs at work [39].

Findings showed associations between increased violence and alcohol use of partners during sex. Alcohol use by both partners in a relationship has been linked to intimate partner violence among all women again irrespective of sex work [6, 14, 22, 23, 40]. In addition to the intervention needing to address signs of alcohol misuse, other services including health or drug and alcohol services could provide important entry points to refer people who may be at risk of IPV. Only 7% of respondents reported that their intimate partner frequently used alcohol, but interventions that try to address and change cultural norms supportive of excessive alcohol use might also be expected to have knock-on effects in terms of primary violence prevention.

## Limitations

As the data are cross-sectional we cannot establish causality or temporal associations between determinants and intimate partner violence. Additionally, in order to increase accuracy in reporting there was some inconsistency in time frames used (i.e. for sexual behaviours, consistent condom use). As a consequence some of the associations could be bi-directional. The use of the WHO tool to measure violence is a strength as this has been validated in other contexts and populations and facilitates comparison [27]. Due to the sensitive nature of questions asked about intimate relationships, there may be underreporting of behaviours, particularly of experiences of intimate partner violence. We attempted to reduce misclassification in behavioural data by our use of female interviewers who conducted interviews in locations selected by participants to minimise their discomfort, embarrassment or fears over disclosure and potential consequences. Although we did not use standardized measures to assess alcohol use among intimate partners, measures were developed through linked qualitative work, to ensure they are appropriate and grounded in the local context and language. Our definition of regular client versus intimate partner may be weakened by the fluid nature of this relationship, with regular clients often moving to the status of intimate partner and back, possibly resulting in misclassification of violence from intimate partners.

## Conclusions

Findings of the study clearly point to the high levels of IPV among female sex workers that needs to be addressed urgently and the association between intimate partner violence and violence from clients, increasing vulnerability of this highly marginalized population. Findings support the need for a multi-level approach to reduce violence including decreasing gender inequalities through education of women, providing community level support structures to address the immediate impacts of violence (including support through criminal justice procedures, immediate medical care and emotional support) and harm reduction interventions to addressing alcohol misuse. The intervention ‘Samvedena Plus’ is designed to do this. Findings are important in that they go some way to dispel misconceptions of female sex workers intimate relationships: trust and demonstrating social support are linked to reduced violence within these relationships; factors common across all types of long-term relationships.

## Abbreviations

AOR: Adjusted odds ratio
CBO: Community based organisation
CI: Confidence interval
DFID: Department for International Development
FSW: Female sex worker
HIV: Human Immunodeficiency Virus
IP: Intimate partner
IPV: Intimate partner violence
IQR: Interquartile range
KHPT: Karnataka Health Promotion Trust
OR: Odds ratio
STI: Sexually transmitted infection
UoM: University of Manitoba
WHO: World Health Organisation
